# The effect of intermittent fasting on microbiota as a therapeutic approach in obesity

**DOI:** 10.3389/fnut.2024.1393292

**Published:** 2024-04-25

**Authors:** Santiago Cadena-Ullauri, Patricia Guevara-Ramírez, Viviana A. Ruiz-Pozo, Rafael Tamayo-Trujillo, Elius Paz-Cruz, Rayner Zambrano-Villacres, Daniel Simancas-Racines, Ana Karina Zambrano

**Affiliations:** ^1^Centro de Investigación Genética y Genómica, Facultad de Ciencias de la Salud Eugenio Espejo, Universidad UTE, Quito, Ecuador; ^2^Universidad Espíritu Santo, Samborondón, Ecuador; ^3^Centro de Investigación de Salud Pública y Epidemiología Clínica (CISPEC), Universidad UTE, Quito, Ecuador

**Keywords:** obesity, microbiota, intermittent fasting, diet, dietary habits

## Abstract

Obesity, a public health challenge, arises from a complex interplay of factors such as dietary habits and genetic predisposition. Alterations in gut microbiota, characterized by an imbalance between Firmicutes and Bacteroidetes, further exacerbate metabolic dysregulation, promoting inflammation and metabolic disturbances. Intermittent fasting (IF) emerges as a promising dietary strategy showing efficacy in weight management and favoring fat utilization. Studies have used mice as animal models to demonstrate the impact of IF on gut microbiota composition, highlighting enhanced metabolism and reduced inflammation. In humans, preliminary evidence suggests that IF promotes a healthy microbiota profile, with increased richness and abundance of beneficial bacterial strains like *Lactobacillus* and *Akkermansia*. However, further clinical trials are necessary to validate these findings and elucidate the long-term effects of IF on microbiota and obesity. Future research should focus on specific tissues and cells, the use of advanced -omics techniques, and exploring the interaction of IF with other dietary patterns, to analyze microbiota composition, gene expression, and potential synergistic effects for enhanced metabolic health. While preliminary evidence supports the potential benefits of IF in obesity management and microbiota regulation, further research with diverse populations and robust methodologies is necessary to understand its implications and optimize personalized dietary interventions. This review explores the potential impact of IF on gut microbiota and its intricate relationship with obesity. Specifically, we will focus on elucidating the underlying mechanisms through which IF affects microbiota composition, as well as its subsequent effects on obesity.

## Introduction

Obesity is a chronic disease that arises from an imbalance in energy between energy intake and expenditure, influenced by behaviors such as eating patterns and physical activity, along with physiological factors like resting metabolic rates and energy expenditure during activity (1, 2). The World Health Organization (WHO) utilizes body mass index (BMI) to classify obesity in adults. For instance, individuals with a BMI >30 kg/m^2^ are considered obese. The classification is further subdivided into three classes. Class I individuals have a BMI between 30.0–34.9 kg/m2; Class II, between 35.0–39.9 kg/m^2^; and Class III, a BMI of more than or equal to 40 kg/m^2^ (3, 4). Class II/III obesity presents higher risks of all-cause mortality, severe health effects, and limits in daily living activities when compared to class I obesity. People with class I obesity are more likely to experience conditions such as hypertension and diabetes mellitus type 2 (5). Class II obesity further increases the risk of secondary diseases such as heart attacks and strokes, aggravating difficulties in performing some actions. Meanwhile, the likelihood of developing secondary conditions is at high risk in type III obesity, often accompanied by symptoms such as severe joint pain, excessive sweating, and breathing difficulties (5, 6).

Furthermore, obesity is a public health challenge for societies and healthcare systems across the world (7). It is one of the main risk factors for several chronic diseases, including gout, osteoarthritis, hypertension, coronary heart disease, stroke, certain cancers, type 2 diabetes, gallbladder disease, and pulmonary diseases (3, 8). In 2016, the estimated prevalence of obesity was 13% (11% of men and 15% of women) (9). WHO estimates that approximately 167 million people will be overweight or obese by 2025 (10). The rising rates and global prevalence of obesity are primarily due to sedentary lifestyles, excessive nutrition, and physical inactivity.

Nutrition and physical exercise are the primary strategies for preventing and managing obesity and its associated metabolic consequences (11). For instance, intermittent energy restriction combined with a Mediterranean diet has emerged as a promising approach to reducing body fat and improving insulin resistance. A recent pilot study conducted among East Asians in Hawaii demonstrated the feasibility and potential efficacy of this combination in reducing visceral adipose tissue (VAT). In this study, participants who followed a Mediterranean diet combined with intermittent fasting (IF) experienced significantly greater reductions in VAT and total fat mass (12).

Understanding the mechanisms behind obesity and the potential impact of interventions like IF is crucial for addressing this global health challenge. This comprehension allows for the development of targeted and effective strategies to prevent and manage obesity-related complications. It also provides insights into how lifestyle modifications, such as dietary interventions, can positively influence insulin sensitivity, fat and glucose metabolic health and overall well-being (13).

Intermittent fasting has attracted substantial scientific and public interest as a dietary strategy for combating obesity (14). IF includes periods of regular caloric intake alternated with complete or partial voluntary abstinence from food and liquid intake (14, 15). There are various IF patterns; the most common are the daily time-restricted fasting (16-h fasting and 8-h eating windows) or the 5:2 diet 2 days of fasting per week and unrestricted eating for the remaining 5 days (15, 16).

The gut microbiota, comprising trillions of microorganisms, produces different physiologically active substances, including short-chain fatty acids and vitamins, as well as potentially harmful products such as neurotoxins and carcinogens (17). A healthy gut microbiota is vital for maintaining metabolic balance and immune function, but dysbiosis can contribute to metabolic disorders and obesity (18). In obesity, alterations in gut microbiota composition can lead to reduced diversity, impacting metabolic energy utilization. For instance, dysbiosis can alter commensal bacteria and their metabolites within the intestinal environment, affecting T cell development and immune responses and causing pro- and anti-inflammatory reactions (19, 20).

Therefore, this review aims to provide an overview of research investigating the influence of intermittent fasting on the gut microbiota and its association with obesity. Our focus will be on elucidating the underlying mechanisms through which IF affects microbiota composition, as well as its subsequent effects on obesity.

### Intermittent fasting

Intermittent fasting (IF) is a dietary strategy defined as intermittent periods of fasting and feeding (21). Time-restricted feeding (TRF), alternate-day fasting (ADF), and the 5:2 diet are the most popular types of IF. TRF is a dietary regimen that limits the feeding time window within a 24-h period. The eating window in TRF ranges from 4 to 12 h, providing flexibility in individual eating patterns (22).

ADF involves alternating between “fast days,” where individuals consume only 25% of their energy needs, and “feed days,” where they eat freely and to appetite. This approach, known for its flexibility, offers an effective weight loss alternative (23, 24). These approaches result in a 1–12% weight reduction over 2–12 months. Moreover, the 5:2 diet involves restricting calorie consumption on two non-consecutive days per week, while on the remaining 5 days, a usual diet is consumed (25).

Furthermore, following alternate feeding and fasting cycles in line with the circadian rhythm, such as eating during the day and prolonging the fasting period overnight, might improve nutrient metabolism. This method allows people to eat freely (with no limitations) during the feeding window, reducing the need to rigorously watch calorie intake outside of the fasting phase (26, 27).

IF triggers several physiological changes within the body, altering metabolism through enzymatic processes in the liver, which causes a drop in insulin levels, increasing glucagon release. These processes cause a shift from glucose to stored fats as energy sources (28, 29). Furthermore, fasting lowers circulating glucose levels by depleting glycogen reserves, resulting in the production of ketone bodies from fatty acids in the liver, which provides an alternate fuel source for many organs, including the brain. Fasting also stimulates autophagy, a cellular recycling mechanism that helps eliminate damaged organelles and proteins, boosting cellular health and lifespan. These physiological modifications help to increase metabolic flexibility and energy consumption during fasting (30, 31).

In obesity, various cellular and molecular processes induce inflammation, especially in adipose tissues, which leads to the release of inflammatory mediators like tumor necrosis factor α (TNF-α), C-reactive protein (CRP), and interleukin 6 (IL-6). Obesity also reduces adiponectin production, leading to a pro-inflammatory state and oxidative stress (32). The activation of NF-κB pathways induces the production of several pro-inflammatory cytokines in adipocytes, which contribute to insulin resistance and pro-inflammatory macrophages. Visceral adipose tissue growth further promotes macrophage recruitment and secretion of inflammatory markers such as CRP, TNF-alpha, and IL-6. Thus, reducing visceral fat through weight reduction may aid in lowering systemic inflammation (33–35).

IF has received attention for its potential benefits in weight control and metabolic health. This dietary approach triggers changes in hormone levels and initiates a metabolic switch, transitioning the body from utilizing glucose as a fuel source to fatty acid-derived ketones. The metabolic switch occurs once liver glycogen stores are depleted, typically beyond 12 h after food intake cessation. This evolutionary trigger shifts metabolism from lipid/cholesterol synthesis and fat storage to fat mobilization through fatty acid oxidation and the production of fatty acid-derived ketones, preserving muscle mass and function (21, 28).

IF also increases the synthesis of adiponectin, a hormone that regulates glucose and breaks down fatty acids (36). Furthermore, IF induces cellular and mitochondrial changes, increasing mitochondrial performance and efficiency. Moreover, IF can potentially influence gene expression and signaling pathways associated with metabolism, inflammation, and oxidative stress, impacting metabolic health and disease risk (28, 31). These processes demonstrate intermittent fasting’s diverse influence on weight control and metabolic balance, indicating its potential as a therapeutic tool for improving health outcomes.

Intermittent fasting, while potentially beneficial, has certain risks and contraindications. For example, IF is not recommended for pregnant or breastfeeding women, frail older adults, individuals with compromised immunity, or people with or at risk for eating disorders due to potential negative health consequences. Moreover, people with diabetes may be more likely to experience hypoglycemia (low blood sugar) during fasting. Additionally, several medications can interact negatively with fasting, posing a risk to individuals with specific medical conditions who require regular medication intake (28).

A study examining 147 individuals with a high BMI following an intermittent fasting regimen revealed common adverse effects, including headache (61.3%), lethargy (68%), mood changes (57.8%), and dizziness and polyuria (55.8 and 46.2%, respectively) (37). Headache, a prevalent side effect during fasting, is often attributed to hypoglycemia and manifests as a diffuse, non-pulsating headache. It is crucial to acknowledge these potential risks and side effects associated with intermittent fasting to ensure safe and informed implementation of this dietary approach (37, 38). On the other hand, various studies have evaluated the benefits of intermittent fasting and have found significant results, including reductions in glucose and insulin levels, as well as notable weight loss and decreased BMI (39–41).

### Microbiota and obesity

Gut bacteria have an important role in the development and progression of obesity. The human gut microbiota is a complex ecosystem that contains around 10^14^ bacterial cells. The diversity of the gut microbiota can influence the human body’s capacity to obtain nutrients and control energy consumption. Humanized mouse models have been useful in understanding the role of the microbiota in obesity, given that they provide a controlled environment to study the interactions between the human intestinal microbiota and host physiology. These models allow researchers to introduce human microbiota into mice, allowing us to observe how specific microbial compositions influence various metabolic processes and contribute to the development of obesity (42). Studies have consistently shown alterations in the composition of gut bacteria in obese individuals, with an increased abundance of Firmicutes and a decreased abundance of Bacteroidetes at the phylum level. Although findings regarding this imbalance may vary across studies, a pattern emerges regarding the diversity of Firmicutes and Bacteroidetes (43, 44). Specifically, the reduction of Bacteroidetes has been linked to fat loss, while the increase in Firmicutes is associated with higher digestible energy intake and fat storage (42, 45).

The gut microbiota profoundly influences energy homeostasis, inflammation, and insulin sensitivity through intricate mechanisms (46). Dysbiosis changes in the gastrointestinal tract, leading to increased gut permeability, result in elevated translocation of bacterial endotoxins, primarily lipopolysaccharide (LPS), into the bloodstream. Activation of the innate immune system via Toll-like receptor 4 (TLR4) by LPS triggers the expression of proinflammatory cytokines, fostering low-grade systemic inflammation associated with insulin resistance, hyperglycemia, and hyperinsulinemia, especially observed in individuals with obesity and type 2 diabetes (47, 48).

**Figure 1:**
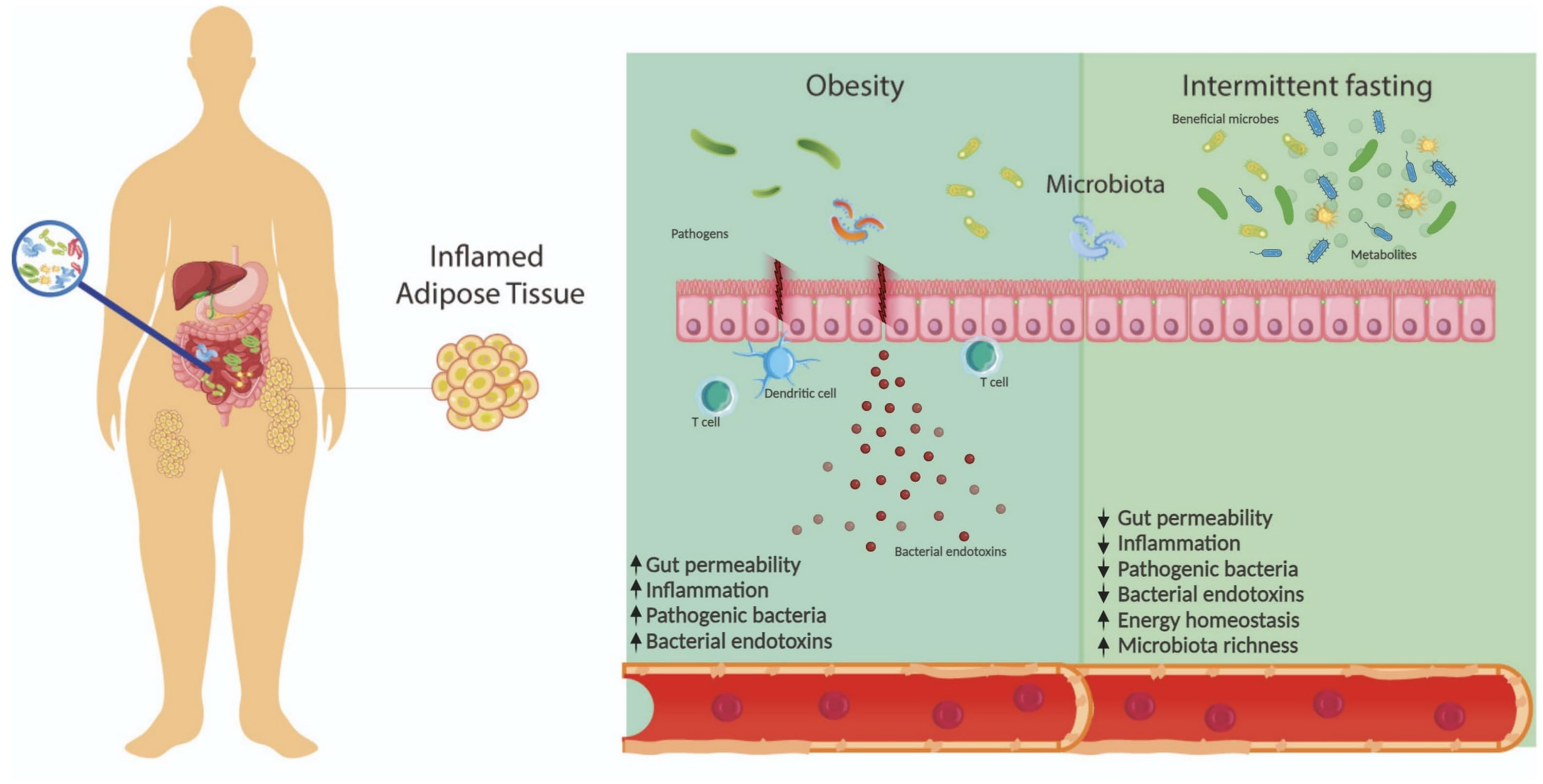
Gut microbiota is influenced by various factors, including diet. In this context, gut microbiota composition is dysregulated in patients with obesity, affecting other processes such as inflammation and gut permeability. Intermittent fasting is a promising approach for weight management and has shown potential benefits to the gut microbiota, including increased microbiota richness and energy homeostasis.

Furthermore, the gut microbiota actively participates in carbohydrate metabolism by fermenting polysaccharides from food, generating monosaccharides and short-chain fatty acids (SCFAs) (49). The gut microbiota influences energy metabolism by modulating the production of SCFAs, which act through various receptors in different tissues, including adipose tissue and the colon. SCFAs improve glucose homeostasis, insulin sensitivity, and gut barrier integrity, attenuating inflammation and promoting metabolic health. Additionally, the gut microbiota synthesizes branched-chain amino acids (BCAAs) and regulates bile acid metabolism, both of which impact insulin resistance and lipid metabolism (47, 50). Overall, the gut microbiota is a dynamic ecosystem with profound implications for host physiology and metabolic health (Figure 1).

### Interplay between intermittent fasting, microbiota, and obesity

A balanced and healthy microbiota maintains a stable ratio of Bacteroidetes to Firmicutes, promoting the production of beneficial metabolites (51). Conversely, individuals with obesity often show imbalances characterized by an increased abundance of Firmicutes and a reduction in Bacteroidetes (51). Moreover, research has found a correlation between the presence of LPS and LPS-producing bacteria with obesity (52). The functionality of the gut microbiota depends on various aspects, including the mode of delivery at birth, medication use, genetics, ethnicity, and dietary habits (53).

Different dietary patterns may favor or affect microbiota homeostasis, influencing the production of metabolites, especially SCFAs, which can participate in various metabolic processes by interacting with endocrine hormones and cell receptors (51). For instance, studies have shown that propionate, a SCFA produced by gut microbiota, can stimulate the release of glucagon-like peptide 1 (GLP-1), thereby promoting weight reduction in overweight adults (54).

### Using mice as a model organism

Intermittent fasting has been proposed as a potential dietary strategy for weight management; however, the effect on microbiota and how it is associated with obesity needs to be further elucidated. Several studies have used mice as model organisms to study this correlation. For example, Beli et al. (55) analyzed the effects of IF on *db/db* mice, commonly used as model organisms for diabetes type II and obesity (56). The research team determined that the gut microbiota of the *db/db* mice was modified by following an IF dietary pattern for 7 months. Specifically, there was an increase in the abundance of Firmicutes, alongside a decrease in the proportion of Bacteroidetes and Verrucomicrobia. Furthermore, *Lactobacillus,* a health-associated genus, was enriched in the IF *db/db* mice (55). These findings have been associated with the concept of ‘healthy obesity’, which is correlated with metabolic health, and an enhanced capacity for energy harvest (57). It is noteworthy that the body weight of the mice did not increase during the study (55).

Furthermore, Li et al. (58) investigated the effects of IF on mice gut microbiota and its association with obesity. The authors found that an IF regimen activated white adipose tissue browning, which mitigated the effects of obesity. The gut microbiota of these IF mice showed an increased abundance of Firmicutes, particularly *Lactobacillus*; while exhibiting a decrease in the proportion of Tenericutes, Actinobacteria, and Bacteroidetes. Moreover, the IF treatment also led to higher levels of acetate and lactate. Interestingly, transplanting the microbiota of IF mice into microbiota-depleted mice also activated white adipose tissue browning (58).

Additionally, Li et al. (59) explored the effects of IF on mice gut microbiota. In this study, the authors found that IF altered the gut microbiota, even in different fasting periods. The results showed decreased levels of *Alistipes* and increased levels of *Akkermansia* (59). *Akkermansia* has been associated with reduced triglyceride accumulation and inflammation (60), whereas *Alistipes* has been correlated with increased inflammation (61). Remarkably, all mice involved in the study experienced reductions in body weight (59).

### Clinical research

Intermittent fasting is a dietary pattern that has been associated with a reduction in obesity in humans. Furthermore, IF has been correlated with a healthy microbiota profile and the production of beneficial metabolites, including lactate and acetate (51). For instance, Cignarella et al. (62) found that IF promoted gut microbiota richness, particularly favoring the presence of *Lactobacillus* and *Akkermansia municiphila*, which have been linked to positive effects on metabolic disorders, including obesity. Interestingly, the authors concluded that IF may modulate immune response by interacting with the microbiota, especially given the increased abundance of *Lactobacilli*, which has been described as having immunomodulatory properties (62).

Similarly, Guo et al. (63) investigated the impact of IF on the microbiota of patients with metabolic syndrome and central obesity. The research team observed an increase in the abundance of the *Rumonococcaceae* family, as well as the genera *Clostridium* and *Roseburia*, further augmenting the proportion of Firmicutes. The *Roseburia* genus has been associated with positive effects on health, such as intestinal inflammation reduction, energy homeostasis maintenance, and immune system maturation (63, 64). Furthermore, Spearman correlation analyses revealed an association between IF gut microbiota and lipid profiles (63). Notably, the authors concluded that IF had a positive impact on gut microbiota and microbial-derived metabolites, ultimately leading to improvements in some aspects of cardiometabolic health (63).

Conversely, Gabel et al. (65) studied the effects of IF on the gut microbiota of adults with obesity. The participants underwent a daily 8-h time-restricted feeding intervention for 12 weeks. Although no significant changes were observed in the microbiota compared to baseline analyses, the participants experienced reductions in body weight (65). Interestingly, the authors suggested that the lack of microbiota changes might be attributed to the specific type of IF diet or to the relative smaller weight reduction compared with previous studies. Table 1 describes the studies mentioned in the present mini-review.

**Table 1:** Gut microbiota differential changes depending on condition.

Condition	Organism	Bacteria	Abundance	Outcome	Reference
IF	*db/db* mice	Firmicutes Bacteroidetes Verrucomicrobia	Increased Decreased Decreased	These results have been associated with the concept of healthy obesity and an enhanced capacity for energy harvest	Xiao et al. (57)
IF	Mice	Firmicutes *Lactobacillus* Tenericutes Actinobacteria Bacteroidetes	Increased Increased Decreased Decreased Decreased	Activation of white adipose tissue browning	Li et al. (58).
IF	Mice	*Alistipes Akkermansia*	Decreased Increased	*Akkermansia* has been associated with reduced triglyceride accumulation and inflammation, whereas *Alistipes* has been correlated with increased inflammation	Li et al. (59)
IF	Human	*Lactobacillus Akkermansia municiphila*	Increased Increased	Positive effects on metabolic disorders, including obesity	Cignarella et al. (62)
IF	Human	*Rumonococcaceae Clostridium Roseburia*	Increased Increased Increased	Positive effects on health, such as intestinal inflammation reduction, energy homeostasis maintenance, and immune system maturation	Guo et al. (63)
IF	Human	No changes	No changes	Weight reduction	Gabel et al. (65).

The molecular mechanisms underlying the beneficial effects of IF on gut microbiota and obesity need to be further elucidated. It has been described that IF induces a metabolic shift from glucose to ketones, thereby promoting ketogenesis. Notably, ketones are synthesized in the liver from fatty acids, further stimulating fat breakdown (66, 67). Furthermore, research has found that bacteria from the *Lactobacillaceae* family can enhance the expression of the fasting-induced adipocyte factor (FIAF), which inhibits lipoprotein lipase (LPL). This inhibition prevents the conversion from triglycerides to fat, regulating lipid metabolism and having a protective effect against obesity (68).

### Clinical implications

Obesity, gut microbiota, and IF constitute a complex network of interactions with significant clinical implications. In this context, obesity can alter gut microbiota, which in turn, may trigger inflammatory cascades and release metabolites that exacerbate obesity-related complications. However, IF emerges as a potential alternative capable of addressing both obesity and gut microbiota dysbiosis (69, 70). Research the potential benefits of IF, including enhanced gut microbial richness and increased abundance of beneficial bacteria such as *Lactobacilli*. Moreover, IF has also been associated with weight loss and improvements in various obesity-related markers (58, 62, 63). Thus, IF represents a valuable tool for promoting metabolic health and fostering a balanced microbiota.

### Limitations

One of the primary limitations in understanding the effects of IF on obesity and microbiota is that most studies have been performed on Western populations (71, 72). Research has suggested that ethnicity notably influences gut microbiota, with studies showing significant variations in gut microbiota as early as 3 months of age (73). Additional limitations include that many studies have been performed with relatively small sample sizes. Research involving larger and more diverse groups of participants is fundamental to comprehensively understanding the impact of IF on microbiota and obesity. Moreover, individual differences between participants in terms of metabolic rate, sex, food preferences, BMI, socioeconomic status, and fitness levels could influence the studies’ results, further complicating analyzing the data. Nutritional interventions present further challenges, as they require modifying the participants’ lifestyle and control over various variables such as food choices and cooking methods. These factors can significantly impact study results. Additionally, this type of research generally includes the use of food records, which may be burdensome for participants (74).

### Future directions

The potential of IF to modulate gut microbiota has shown promise as a way to manage weight and regulate gut microbiota. A bibliographic search conducted on the database ClinicalTrials.gov, an official website of the U.S. Department of Health and Human Services, National Institutes of Health, National Library of Medicine, and National Center for Biotechnology Information, using keywords such as “Obesity” for Condition/disease, “Microbiota” for Other terms, and “Intermittent fasting” for Intervention/treatment, revealed that there are currently seven studies involving these search terms. However, no results have been posted for any of these studies to date. Therefore, further research is crucial to fully understand the impact of IF on gut microbiota and obesity (75).

Future research on IF could explore the diverse variations of the IF diet. For instance, studies should investigate different fasting schedules, such as Time-Restricted Feeding and Alternate-Day Fasting, to determine the effects of each regimen on obesity and gut microbiota composition.

Moreover, determining the impact of IF on specific tissues and types of cells is crucial to comprehend its molecular mechanisms. For example, liver cells regulate metabolic processes, whereas adipose tissue cells have a key role in energy homeostasis; therefore, the effects that IF will have on overall health will depend on how IF interacts with each particular tissue and type of cell.

Furthermore, incorporating the use of innovative -omics techniques that provide a comprehensive analysis of biological systems at various molecular levels, such as genomics, transcriptomics, proteomics, and metabolomics, and can offer a comprehensive perspective on microbiota composition, metabolites, and gene expression patterns in response to IF. These technologies enable the identification of novel biomarkers, pathways, and therapeutic targets against obesity. Additionally, they could pave the way for targeted interventions and personalized approaches to weight management.

Lastly, analyzing the interplay of IF with other diets could yield valuable information, which can lead to the development of customized dietary patterns tailored to specific health goals. For instance, understanding how IF interacts with diets, such as the Mediterranean, could reveal synergistic effects that improve gut microbiota composition, metabolic health, and weight management.

In conclusion, research has shown that IF is a dietary pattern with the potential to positively influence human health by interacting with gut microbiota and ameliorating the effects of obesity. While existing research suggests a beneficial impact of IF on human health, including improved metabolic health and weight management, further studies are required to improve our understanding of this interaction. It is important to highlight that these studies should improve data analysis and collection, include diverse populations, and determine the molecular mechanisms involved.

## Author contributions

SC-U: Conceptualization, Investigation, Writing – original draft, Writing – review & editing. PG-R: Conceptualization, Investigation, Writing – original draft, Writing – review & editing. VR-P: Investigation, Writing – review & editing. RT-T: Investigation, Writing – review & editing. EP-C: Investigation, Writing – review & editing. RZ-V: Investigation, Writing – review & editing. DS-R: Investigation, Writing – review & editing. AZ: Conceptualization, Investigation, Supervision, Writing – review & editing.
